# Internal carotid artery aneurysm with incomplete isolated oculomotor nerve palsy: a case report

**DOI:** 10.1186/s13256-023-03758-8

**Published:** 2023-02-20

**Authors:** Jing Zheng, Yue Wan

**Affiliations:** The Third People’s Hospital of Hubei Province, Wuhan, Hubei China

**Keywords:** Aneurysm, Internal carotid artery, Oculomotor nerve palsy

## Abstract

**Background:**

Oculomotor nerve palsy is a common and well-described disease with diverse etiologies. Clinicians should quickly and correctly diagnose and treat oculomotor nerve palsy according to its characteristics and the accompanying symptoms and signs. Intracranial aneurysm is an important and frequent cause of oculomotor nerve palsy. Considering the catastrophic consequences of rupture, the possibility of an urgent, life-threatening disease should always be considered.

**Case presentation:**

A 63-year-old Chinese woman presented with intermittent left ptosis and diplopia and painless incomplete oculomotor nerve palsy without pupil involvement. She manifested no mydriasis or extraocular muscle weakness, and the light reflex was normal. Other cranial nerves and somatosensory and somatomotor examinations were normal. The neostigmine experiment and electromyography were normal, so the diagnosis of myasthenia gravis was excluded. Brain magnetic resonance angiography showed a 4-mm aneurysm located at the cavernous segment of the left internal carotid artery. Unfortunately, the patient refused digital subtraction angiography and was discharged home without further treatment.

**Conclusion:**

Neuroimaging must be performed to exclude intracranial aneurysms in oculomotor nerve palsy regardless of whether the pupils are involved, as aneurysm rupture carries substantial morbidity and mortality.

## Introduction

Oculomotor nerve palsy is a common and important neurological disease that classically manifests with ptosis, diplopia and extraocular muscle weakness. The presenting clinical symptoms and signs of oculomotor nerve palsy vary according to the etiologies and locations of the lesion. Oculomotor nerve palsy can result from intracranial aneurysm, trauma, diabetes mellitus, tumors, stroke, and infection. Due to the complexity of its etiologies, comprehensive neurological examinations, blood tests, neuroimaging, and lumbar puncture should be performed to help quickly and correctly diagnose oculomotor nerve palsy. It is also very important to distinguish isolated oculomotor nerve palsy (no other neurological symptoms or signs) from those that are not isolated. An ischemic neuropathy and a compressive lesion are leading causes of isolated oculomotor nerve palsy [1]. Traditional teaching is that the pupil is dilated and poorly reacted to light when an aneurysm compresses the oculomotor nerve, and the pupillary sphincter is spared in ischemic injury [2]. Herein, we report a case of pupil-sparing isolated oculomotor nerve palsy attributed to a left internal carotid artery aneurysm in the left cavernous sinus.

## Case report

**Fig. 1:**
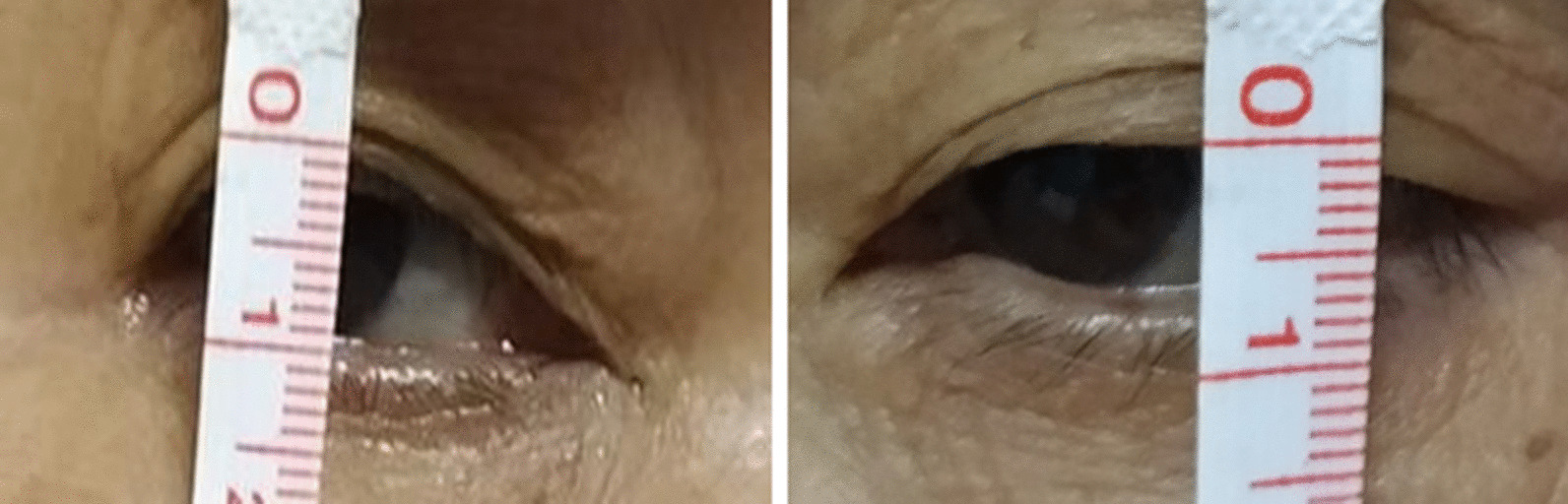
Left ptosis of the patient

**Fig. 2:**
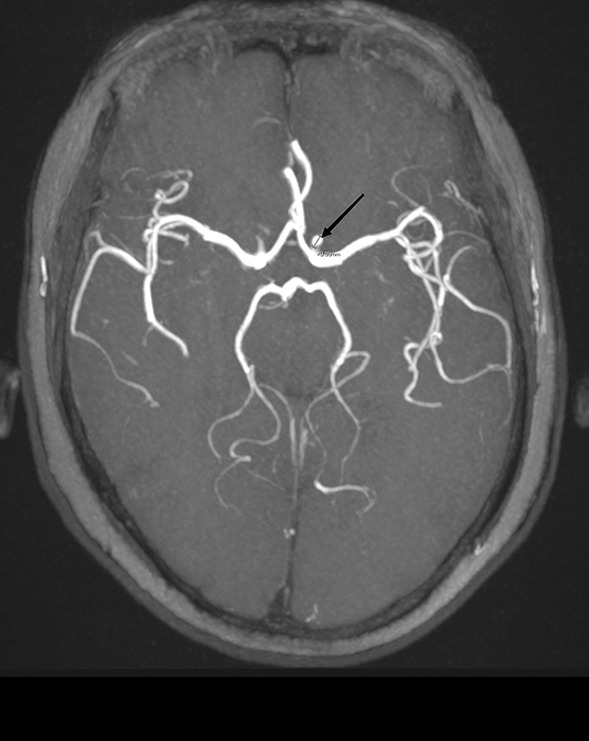
Magnetic resonance angiography showing a 4-mm aneurysm located at the cavernous segment of the left internal carotid artery (black arrow)

**Fig. 3:**
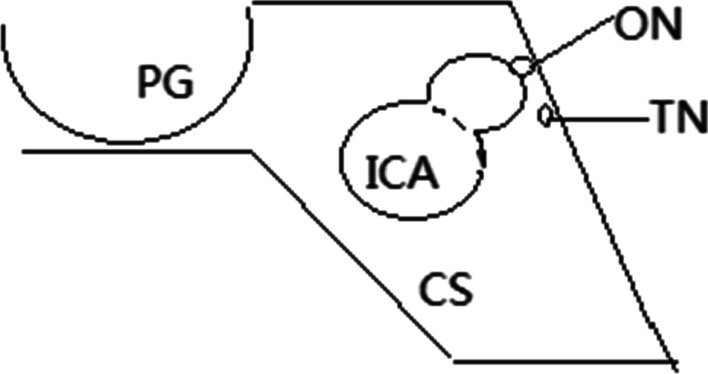
Simple sketch demonstrating the relationship of the aneurysm to the oculomotor nerve. *PG* pituitary gland, *TN* trochlear nerve, *CS* cavernous sinus

A 63-year-old Chinese woman presented with intermittent left ptosis and diplopia beginning half a year ago (Fig. 1). The ptosis fluctuated and increased as the day progressed. She denied having any headache. The ptosis intensified, prompting her to see the eye doctor. Ophthalmologic examination was normal, so she was transferred to the neurology clinic based on the suspicion of myasthenia gravis. She was on no medications and had no significant medical history. Her neurological examination was remarkable only for left-sided ptosis. She manifested no mydriasis or extraocular muscle weakness, and the light reflex was normal. Other cranial nerves and somatosensory and somatomotor examinations were normal. We have performed an extensive diagnostic workup to determine the etiology since her admission. White blood cell count was 3.18 × 10^9^/L, but thrombocytes and red blood cells were normal. Electrolytes, hepatic tests, renal tests, erythrocyte sedimentation rate, vitamin B12, and thyroid tests were normal. Systematic autoimmune autoantibody testing was unrevealing. Blood testing of treponemal results with rapid plasma reagin (RPR) was negative. The initial diagnosis of ocular myasthenia gravis was made on the basis of intermittent and progressive ptosis. Pyridostigmine bromide (720 mg/day) was started, but her symptoms did not resolve. The neostigmine experiment and electromyography were normal, so the diagnosis of myasthenia gravis was ultimately ruled out. Brain magnetic resonance imaging (MRI) was unremarkable, but magnetic resonance angiography (MRA) showed a 4-mm aneurysm located at the cavernous segment of the left internal carotid artery (Figs. 2, 3). Regretfully, the patient refused cerebral digital subtraction angiography and was discharged home without further treatment.

## Discussion

The oculomotor nerve nucleus is located in the dorsal midbrain. The oculomotor nerve fascicle tracks through the red nucleus, exits ventrally, passes between the posterior communicating artery and the superior cerebellar artery, lies in a dural fold along the superolateral wall, and heads toward the superior orbital fissure to innervate the extraocular muscles, the iris sphincter muscle, and the elevator palpebrae superioris muscle [3]. Any lesions located from the oculomotor nucleus to the termination oculomotor fascicles can lead to oculomotor nerve palsy. Intracranial aneurysm is a common cause of oculomotor nerve palsy, and the prevalence of aneurysm as a cause of isolated oculomotor nerve palsy has been reported to range from 34% to 56% [4]. Mechanical deformation, pressure variation, and nerve edema produced by acute expansion of the unruptured aneurysm sac can cause oculomotor nerve palsy [5, 6]. The posterior communicating artery and basilar artery aneurysms causing isolated oculomotor nerve palsy were reported to be ≥ 4 mm [7].

The Rule of the Pupil states that, when an aneurysm compresses the oculomotor nerve, the iris sphincter will be impaired, and the affected pupil will be dilated or sluggishly reactive to light [8]. Walsh and Hoyt stated that “in most cases of pupil-sparing oculomotor palsy, the patient suffers from diabetes” [8]. A more than 75% probability of pupil-sparing palsy is attributed to ischemic microvascular disease, a 4% probability is attributed to aneurysm, and a 2% probability is attributed to tumor [10]. The pupillomotor fibers reside in the superficial and dorsomedial portions of the oculomotor nerve, which are susceptible to impingement from intracranial aneurysms. Reversible neuropraxia is the first reaction of the nerve to compression; if compression persists, microanatomical changes begin until the occurrence of axonal degeneration [11]. Aneurysms at the junction of the internal carotid artery and posterior communicating artery cause compression of the superomedial pupil fiber first and lead to pupil involvement oculomotor nerve palsy. This explains the symptom of pupil dilation in most cases of aneurysmal oculomotor nerve palsy. The “rule” is relatively simple and does not apply when oculomotor nerve functions are partially rather than completely lost. Rare cases of pupil-sparing incomplete oculomotor nerve palsy caused by an intracranial aneurysm have been described [12, 13]. The different growth directions and pressure equilibriums of aneurysms or the pupillomotor fibers traveling in the lateral direction of the oculomotor nerve result in the noncompression of the pupillomotor fibers. Kerr and Hollowel demonstrated that a possible anatomical explanation for pupillary sparing is that, after compression of the dorsomedial pupillomotor fibers due to a lesion, the supplementary parasympathetic fibers running laterally in the nerve may be sufficient to maintain the pupillary tone [11]. In the cavernous sinus, aneurysm-related compressive lesions have a propensity to spare the inferior portion of the oculomotor nerve, causing ptosis but leaving pupilloconstriction intact [14]. This may explain the pupil-sparing oculomotor nerve palsy in our patient. Our case contradicts the dictum that “if the pupil is spared, the aetiology of the problem is not an aneurysm” [8].

Intermittent ptosis and diplopia were the only neurological signs in our aneurysmal oculomotor nerve palsy. As Jaeger reported previously, mild ptosis with mild mydriasis was the minimum sign of aneurysm-related oculomotor nerve function [15]. Previous literature suggested that at least one of either ptosis, mydriasis, or extraocular muscle weakness was spared in minimal oculomotor nerve paresis due to unruptured aneurysms [15]. The patient denied having any headache or retrobulbar pain, and 68.8% of patients in one series had retrobulbar pain, which has been attributed to the compression of pain sensory afferent fibers from trigeminal fibers within the periphery of the oculomotor nerve [16]. Thus, the presence or absence of pain in association with oculomotor nerve palsy cannot rule out the possibility of aneurysm. The manifestation of intermittent ptosis in our patient mimicked ocular myasthenia gravis, but the inefficiency of pyridostigmine bromide and subsequent normal electromyography tests excluded myasthenia gravis.

## Conclusion

The presentation of our case underlines the existence of pupil-sparing incomplete aneurysmal oculomotor palsy. Aneurysm-related oculomotor nerve palsy is a warning sign of potential aneurysm ruptures. Considering the serious consequences of rupture, regardless of whether the pupil is affected in oculomotor nerve palsy, neuroimaging should be performed as soon as possible to rule out intracranial aneurysm.

## Data Availability

Data sharing is not applicable to this article as no datasets were generated or analyzed during the current study.

## Abbreviations

E‒W: Edinger–Westphal
MRA: Magnetic resonance angiography
DSA: Digital subtraction angiography
